# Exploring the mechanism of action of tripterygium glycosides tablets in the treatment of rheumatoid arthritis based on the Lnc-ENST00000602558/IGF1 signaling pathway

**DOI:** 10.3389/fphar.2026.1864355

**Published:** 2026-08-20

**Authors:** Jin-ying Fang, Ming-xuan Liu, Yan-nan Yang, Cun-xiang Xie, Chun-ping Liu, Chao-hua Zhu, Jie Hu, Wei Li, Liu Lv, Yong-hong Wang, Zheng-hong Zhu, Hai-long Wang

**Affiliations:** Dongzhimen Hospital, Beijing University of Chinese Medicine, Beijing, China

**Keywords:** IGF1, JAK/STAT pathway, Lnc-ENST00000602558, rheumatoid arthritis, Tripterygium glycosides

## Abstract

**Objective:**

Rheumatoid arthritis (RA) is a chronic inflammatory joint disease and the leading cause of joint-related limb disability. Traditional Chinese Medicine (TCM) offers certain advantages in RA treatment, and Tripterygium preparations are widely used due to their significant clinical efficacy. This study aimed to investigate the mechanism of action of Tripterygium Glycosides Tablets (TG) in the treatment of RA through *in vivo* and *ex vivo* experiments, providing a theoretical basis for clinical application.

**Methods:**

A collagen-induced arthritis (CIA) rat model was established, and the rats received oral administration of the corresponding drugs or distilled water for 4 weeks. The therapeutic effect and mechanism of TG on CIA rats were evaluated by comparing joint swelling severity, radiographic changes in the synovium, and alterations in the JAK/STAT signaling pathway and related inflammatory factors. Further *ex vivo* experiments were performed using fibroblast-like synoviocytes (FLS) as the research subject. Lentiviral infection technology was used to overexpress IGF1 or interfere with the expression of Lnc-ENST00000602558. Drug-containing serum was used for intervention. The mechanism of TG in treating RA was further elucidated by detecting changes in cell migration ability, the expression of IGF1 and Lnc-ENST00000602558, JAK/STAT pathway-related targets, and inflammatory factors.

**Results:**

TG significantly improved joint swelling, bone and cartilage destruction in CIA rats, reduced synovial tissue hyperplasia, inhibited the JAK/STAT pathway and IGF1 in the rat joint synovium, lowered serum levels of the inflammatory factors TNF-α and IL-1β, alleviated joint damage, and delayed disease progression. *Ex vivo* experiments showed that the Lnc-ENST00000602558/IGF1 axis could activate the JAK/STAT pathway in FLS. When Lnc-ENST00000602558 was expressed at low levels, the regulatory capacity of TG on the Lnc-ENST00000602558/IGF1 axis and the JAK/STAT pathway was diminished.

**Conclusion:**

TG may regulate the JAK/STAT pathway via the Lnc-ENST00000602558/IGF1 axis, thereby inhibiting the inflammatory response, mitigating bone and cartilage destruction, and delaying disease progression.

## 1 Introduction

Rheumatoid arthritis (RA) is a chronic inflammatory joint disease primarily characterized by joint pain and morning stiffness, with cartilage and bone destruction as its hallmark feature. Statistics indicate that RA patients account for approximately 0.1% of the global population, with an estimated five million patients in China. Notably, RA is the leading cause of joint disease-related limb disability (Wang et al., 2021; Sparks, 2019; Gabriel et al., 2003). A diverse range of therapeutic agents is available for RA, including disease-modifying anti-rheumatic drugs (DMARDs), nonsteroidal anti-inflammatory drugs (NSAIDs), and glucocorticoids. These drugs can effectively alleviate pain and suppress inflammatory responses in patients with RA. However, their clinical application is limited by severe hepatorenal toxicity, gastrointestinal damage, and other adverse effects, leaving over 50% of patients without satisfactory treatment outcomes (Bindu et al., 2020; Conigliaro et al., 2019; Smolen et al., 2016). Traditional Chinese Medicine (TCM) offers certain advantages in RA treatment. Among these, preparations of Tripterygium wilfordii are widely used due to their significant clinical efficacy. Tripterygium glycoside tablets (TG) have demonstrated non-inferior to methotrexate (MTX) and other anti-rheumatic drugs in treating RA, and can exert synergistic effects in combination with them (Lin et al., 2020). Supported by research findings on Tripterygium wilfordii, experts have formulated the “2023 International Guideline for Tripterygium wilfordii in the Treatment of Active RA.” This marks a classic paradigm of the Chinese approach, which integrates integrating TCM and standard biomedical treatment on a global scale (Zhang et al., 2023; Lin et al., 2020; Zhou et al., 2018).

Recent studies have revealed that long non-coding RNAs (lncRNAs) can interact with DNA, RNA, and proteins, serving as potential key regulators in RA (Yang et al., 2022; Jiang et al., 2023). Given the high heterogeneity of RA, a comprehensive assessment of patient characteristics—including disease course, clinical status, genetic background, and epigenetic features—can be achieved by analyzing various lncRNAs (such as HOTAIR, GAS003209, and HIX5) in patient plasma, synovial tissue, and immune cells (Yang et al., 2022; Jumaa et al., 2026). This approach facilitates early diagnosis, stratified management, and rational drug use, thereby improving the rates of treatment target attainment and enabling precision medicine for RA (Wu et al., 2021; Elhai et al., 2023; Kiełbowski et al., 2025).

In our previous study, an integrated strategy combining whole-transcriptome microarray detection and biomolecular network analysis was employed to investigate the lncRNA-mRNA network regulatory mechanisms underlying the individual differences in the efficacy of Tripterygium wilfordii (Gao et al., 2024). Co-expression correlation analyses of lncRNAs and mRNAs were conducted, and a “disease gene-all co-expressed efficacy gene interaction network” was constructed. Results revealed that the most highly interacting co-expressed gene pair in this network was IGF1, which exhibited 17 interactions, including interactions with the JAK/STAT signaling pathway, a known therapeutic target for RA. By querying the Ensembl database, a specific lncRNA-mRNA pair, Lnc-ENST00000602558/IGF1, was identified. A comprehensive literature review revealed no reports, other than those from our research group, on Lnc-ENST00000602558 in the context of Tripterygium wilfordii treatment for RA. Furthermore, peripheral blood mononuclear cells from RA patients who responded well to Tripterygium wilfordii treatment and non-responders were analyzed using Affymetrix, EG 1.0 Array technology. The results indicated that the co-expression of Lnc-ENST00000602558 and IGF1 was associated with TG efficacy in RA treatment. Therefore, the expression levels of genes within the Lnc-ENST00000602558/IGF1 signaling pathway are associated with the effectiveness of TG treatment in RA patients (Gao et al., 2024).

This approach aimed to elucidate the mechanism underlying the efficacy of TG in RA treatment. Although the IGF1 and JAK/STAT signaling pathways are known to play critical roles in the pathogenesis of RA, therapeutic options targeting these pathways remain limited. This study identifies a novel mechanism by which TG suppresses the JAK/STAT signaling pathway through regulation of the Lnc-ENST00000602558/IGF1 axis. This finding offers a new molecular perspective on the therapeutic action of TG.

Experimental models of RA are broadly divided into induced and spontaneous genetically modified types (Pradhan et al., 2026; Su et al., 2026). Each model has its own strengths and limitations (Miyoshi and Liu, 2024), but a key feature of RA pathophysiology is the production of inflammatory cytokines—such as TNF-α, IL-1, and IL-6—by synovial cells. The collagen-induced arthritis (CIA) model effectively captures this process and shares several pathological hallmarks with RA, including synovial hyperplasia, synovitis with cellular infiltration, cartilage destruction, and bone erosion. As a result, the CIA model remains the most widely used experimental system in RA research. It has also been extensively employed to investigate the underlying autoimmune mechanisms, such as the roles of specific cell types in disease onset and progression, and has served as a critical platform for designing and evaluating new therapeutic strategies (Jad et al., 2026).

This study employed CIA rat model to investigate the role of the JAK/STAT pathway in the therapeutic effects of TG on RA. Using lentiviral infection technology (Li et al., 2023), the influences of IGF1 and Lnc-ENST00000602558 on the levels of JAK/STAT pathway-related proteins and inflammatory cytokines were further examined in fibroblast-like synoviocytes (FLS).

## 2 Materials and methods

### 2.1 Materials

#### 2.1.1 Cell

The FLS were isolated from RA patients who underwent total joint arthroplasty or synovectomy. All RA patients met the 1987 Guidelines of the American Rheumatology Association or the 2010 ACR/European League against Rheumatism (EULAR) Criteria. FLS characterization used flow cytometry to detect CD68 and Vimentin in the cells. CD68 was used to characterize the macrophage-like cellular properties of the primary cells, and Vimentin is used to characterize the fibroblast-like properties of the primary cells, refer to Supplementary Figures 5, 6.

#### 2.1.2 Animal

40 male SD rats were 5–6 weeks old which were obtained from SPF (Beijing) Biotechnology Co., Ltd. Beijing, China. Prior to the experiment, all SD rats were acclimated to the laboratory of Beijing University of Chinese Medicine for 1 week. The sample size was calculated using GPower 3.1.9.7 software (Heinrich-Heine-Universität Düsseldorf, Germany). The arthritis index served as the primary endpoint, based on results from our preliminary experiment. The anticipated mean difference between the model group and the TG group was set at 3.0, with a pooled standard deviation of 2.0 (effect size: Cohen’s d = 1.5). An independent t-test was used with a two-sided significance level (α) of 0.05 and statistical power (1−β) of 0.80. This calculation indicated a minimum effective sample size of eight animals per group. To account for an approximately 85% success rate of CIA model induction, the enrollment number was adjusted to 10 animals per group. The CIA model rats were used to simulate the pathological state of RA patients. The modeling method was as follows: (1) Collagen preparation: Under light protection and an ice bath, bovine type II collagen and Fuchs’ incomplete adjuvant were fully emulsified into a water-in-oil mixture at a 1:1 ratio; the drops did not disperse in water. (2) Collagen injection: One week after acclimatization, 0.2 mL of the prepared collagen emulsion was subcutaneously injected into the left tail root of the rats. Seven days after the initial injection, 0.1 mL of the collagen emulsion was injected into the right tail root. The placebo group was injected with saline, and the rest of the procedure was the same as that of the model group.

#### 2.1.3 Preparation of drug-containing serum

Thirty male SD rats were randomly divided into three groups (n = 10 per group): a placebo group, an MTX group, and a TG group. The rats received acclimatization feeding for 3 days prior to being divided into groups. The TG group received 12.6 mg/kg/day, while the MTX group received 3 mg/kg/day. The drugs were dissolved in distilled water and administered to the test groups via tube feeding, while the placebo group received distilled water. On the sixth day of administration, abdominal aorta sampling was performed two to 4 hours after the conclusion of tube feeding. Blood was obtained by centrifugation at 3,000 rpm for 20 min at 4 °C. The sera from each group were mixed together and passed through a 20-μm microporous membrane filter to remove bacteria. The separated sera were stored in a −80 °C refrigerator.

#### 2.1.4 Drug preparation

TG tablets (Zhejiang Derende Pharmaceutical Co., Ltd. Shaoxing, Zhejiang, China, production batch number: 2405107B, and the state pharmaceutical license number: Z33020422). Were crushed into a powder in a mortar and then dissolved in distilled water to prepare reglanoside solvents with a mass concentration of 10 mg/mL. In accordance with the requirements of the Chinese Pharmacopoeia, liquid chromatography-mass spectrometry (LC-MS) was used to detect the main components (Triptolide and Wilforlide A) in the drug; further details are provided in the Supplementary Figures 1–4. MTX tablets (Shanghai Xinyi Pharmaceutical Co., Ltd. Shanghai, China, Batch No.: 197220809; State Pharmaceutical License No.: H31020644) were crushed into a powder using a mortar and pestle. Then, they were dissolved in distilled water and formulated into an MTX solvent with a mass concentration of 5 mg/mL. The drug dosage for rats was determined based on body surface area (BSA) conversion (the Meeh-Rubner formula) and further validated through preliminary experiments. The TG group received 10 mg/kg/d by gavage, the MTX group received 1 mg/kg/w by gavage, and the placebo and model groups received distilled water by gavage.

### 2.2 Observation indices

#### 2.2.1 Cell counting kit-8 (CCK-8) assay

FLS cells were seeded into 10-well plates at 1 × 10^4^ cells per well and cultured in complete medium for 24 h. To study the effect of TG on cell toxicity, serum containing TG, MTX, or placebo was added to the culture medium. After treatment with different serum concentrations (5%, 10%, and 15%) and exposure times (0, 6, 12, 24, 36, 48, and 72 h), 10 μL of CCK-8 reagent was added to each well. The cells were incubated at 37 °C for 1–2 h and absorbance at 450 nm was then using an automated microplate reader. Each measurement was performed at least three times, and all treatments were repeated in triplicate.

To investigate the inhibitory effect of TG tablets on FLS cell proliferation under inflammatory conditions, cells were divided into three groups: placebo, model, and TG. Cells were seeded in 96-well plates at a density of 5 × 10^3^ cells per well and cultured in DMEM at 37 °C for 24 h.

After 24 h, LPS (1 μg/mL) was administered to both the model group and the TG group, while the TG group also received TG-containing serum at various concentrations. The culture was then continued for another 24 h, after which 10 μL of CCK-8 reagent was added per well. Cells were subsequently incubated at 37 °C for 2–3 h. Absorbance at 450 nm was measured using an automated microplate reader. Each measurement was performed at least three times, and each treatment was repeated in triplicate.

#### 2.2.2 FLS scratch experiment

First, a horizontal line was drawn parallel to the transverse axis of the culture plate on the bottom of a 6-well plate using a marker pen and straightedge. This facilitated subsequent positioning and imaging. FLS from passages three through eight with good growth status were selected and seeded into the six-well plate at a density of 2–5 × 10^5^ cells per well following different interventions. The cells were cultured for 24–48 h. Using a straightedge, a perpendicular scratch was made relative to the transverse axis of the culture plate with a pipette tip. The cells were then cultured for 24 h. Images were captured at 0, 12, and 24 h. The scratch area was quantified using ImageJ software.

#### 2.2.3 Western blot (WB)

WB was performed to detect the levels of JAK1, p-JAK1, STAT1, p-STAT1, JAK2, p-JAK2, STAT3, p-STAT3, and IGF-1 in the synovium and FLS of CIA rats. The knee synovium was collected, immediately frozen in liquid nitrogen, and stored at −80 °C. For protein extraction, tissue homogenates were prepared in a lysis buffer containing 1 mM PMSF. To denature the proteins, equal amounts were electrophoresed in 10% bis-Tris/polyacrylamide gels and transferred to PVDF membranes. The membranes were blocked with 5% nonfat milk for 2 hours and incubated overnight at 4 °C with primary antibody (Abcam, Cambridge, United Kingdom) diluted in blocking solution. They were then incubated with a horseradish peroxidase-coupled secondary antibody for 2 h at room temperature, and detect immunoreactivity using enhanced chemiluminescence. Band intensity was measured by scanning the blots and analyzing them using UN-SCAN-IT version 5.1 software.

#### 2.2.4 Dual-luciferase reporter assay

The dual-luciferase reporter assay enables simultaneous expression of two distinct luciferases, thereby improving the accuracy and credibility of experimental data. Wild-type and mutant sequences of IGF1 and ENST00000602558 were cloned into vectors and used to construct reporter gene plasmids. The interaction between IGF1 and ENST00000602558 was then evaluated using this assay.

For this experiment, the relevant plasmids were first constructed. The promoter control plasmid was CON663; the target gene promoter plasmid was PL-IGF1 (K24K0006); the transcription factor control plasmid was CON520, while the transcription factor plasmid was PL-ENST00000602558 (K24K0007). The grouping after transfection is shown in Supplementary Table 1.

#### 2.2.5 Reverse transcription polymerase chain reaction (RT-PCR)

Levels of the Lnc-ENST00000602558 and IGF1 mRNA in cells were measured by real-time quantitative PCR. Total RNA was extracted from the spinal cord tissue by homogenization using the TaKaRa MiniBEST Universal RNA Extraction Kit (TaKaRa, Kusatsu, Japan) under RNase-free conditions, following the manufacturer’s protocol. For reverse transcription, total RNA (1 μg) was converted to cDNA using the PrimeScript™ RT Kit with gDNA Eraser (TaKaRa, Kusatsu, Japan). Total RNA (1 μg) was extracted from the spinal cord using SYBR® Premix Ex Taq™ II (Tli RNase H Plus) and ROX Plus (TaKaRa, Kusatsu, Japan). Specific transcripts were quantified using SYBR® Premix Ex Taq™ II (TliRNaseH Plus) and ROX Plus (TaKaRa, Kusatsu, Japan), and the results were analyzed using an ABI 7500 Real-Time PCR System (Applied Biosystems, Foster City, CA, United States of America). RT-PCR primers were designed by Hunan Acres Bioengineering Co. The PCR program consisted of 95 °C for 30 s, followed by 40 cycles of 95 °C for 5 s, 55 °C for 30 s, and 72 °C for 30 s. Melting curve analysis was performed from 60 °C to 95 °C. Relative gene expression was calculated using the 2^−ΔΔCT^ method. Primer sequences for the Lnc-ENST00000602558 and IGF1, along with the internal reference, are provided in Supplementary Table 2.

#### 2.2.6 Enzyme-linked immunosorbent assay (ELISA) experiment

An ELISA kit was used to measure the protein levels of TNF-α and IL-1β protein expression in the serum and cell supernatant of rats from each group.

#### 2.2.7 Hematoxylin and eosin (HE) staining

Paraffin-embedded sections of rat joints, processed following fixation in 4% paraformaldehyde, embedding, sectioning, and baking, were deparaffinized. They were then stained with hematoxylin and eosin, dehydrated, cleared, and mounted. The stained sections were subsequently examined under a microscope.

#### 2.2.8 Safranin O-fast green staining

Deparaffinized blank sections of rat joints were stained with Fast Green and Safranin O solutions. The sections were then dehydrated, cleared, mounted, and examined under a microscope.

#### 2.2.9 Micro-computed tomography (micro-CT) analysis

The left knee joint, left ankle joint, and left paw of the rats were scanned with Micro-CT (Inveon CT, Siemens, Germany) to generate three-dimensional images of the articular structures and to evaluate bone-related parameters. Quantitative analysis of the images was performed using the Region of Interest (ROI) module in the Inveon Research Workplace software package.

Severity of Ankle Joint Bone Erosion: Micro-CT was used to scan the left knee joint, left ankle joint, and left foot of rats. Bone-related parameters such as Bone Mineral Density (BMD), Bone Volume (BV), Bone Surface (BS), bone surface fraction (BS/BV), Bone Volume/Total Volume (BV/TV), Trabecular Thickness (Tb.Th), Trabecular Number (Tb.N), Trabecular Spacing (Tb.Sp), and Trabecular Pattern Factor (TBPf) were measured.

#### 2.2.10 Immunohistochemistry (IHC)

Joint paraffin sections were deparaffinized with xylene and dehydrated through a graded series of alcohols after incubation at 60 °C for 1 h. Endogenous peroxidase activity was blocked with 3% H_2_O_2_, and heat-induced epitope retrieval was conducted in citrate buffer. Sections were incubated overnight at 4 °C with primary antibodies against JAK1, p-JAK1, STAT1, p-STAT1, JAK2, p-JAK2, STAT3, and p-STAT3. They were then incubated with SignalStain® Boost IHC Detection Reagent (HRP, Rabbit) (Cell Signaling Technology, Danvers, MA, United States of America) according to the manufacturer’s instructions. The final colorimetric product was developed using the SignalStain DAB Substrate Kit (Cell Signaling Technology, Danvers, MA, United States of America), and sections were counterstained with hematoxylin (Leagene, Beijing, China). Images of the ankle joint pathology sections were captured using a microscope equipped with an integrated digital camera system. For images taken at 200× and 400× magnification, semi-quantitative analysis of the protein expression levels of JAK1, p-JAK1, STAT1, p-STAT1, JAK2, p-JAK2, STAT3, and p-STAT3 was performed in 10 consecutive fields using ImageJ software.

#### 2.2.11 Lentiviral infection assay in FLS

The lentiviral vectors were constructed with the assistance of Genomeditech Co., Ltd. To generate the Lnc-ENST00000602558 intervention plasmid (LV-shlnc2558) and the IGF1 overexpression plasmid (LV-pcIGF1), appropriate vectors were employed. FLS cells infected with these constructs are subsequently denoted by these symbols. Lnc-ENST00000602558 refers to cells with normal Lnc-ENST00000602558 expression, and LV-ncIGF1 denotes counterparts with normal IGF1 expression.

### 2.3 Statistical analysis

Statistical analysis was conducted using SPSS 21.0 software, and the figures were generated with GraphPad Prism 9.5.0. Measurement data are expressed as the mean ± standard deviation. A one-way ANOVA test was used to compare means among three or more groups. Count data are expressed as frequency (percentage) [n (%)]. Groups comparisons for categorical variables were performed using the chi-square test (χ^2^ test). Pairwise comparisons of sample rates and group means were conducted using,the Bonferroni correction and LSD-t tests, respectively. All statistical tests were two-sided with a 95% confidence interval, and a P value <0.05 was considered statistically significant.

## 3 Results

### 3.1 Animal experimental results

#### 3.1.1 TG tablets alleviate bone and cartilage damage in CIA SD rats

**FIGURE 1:**
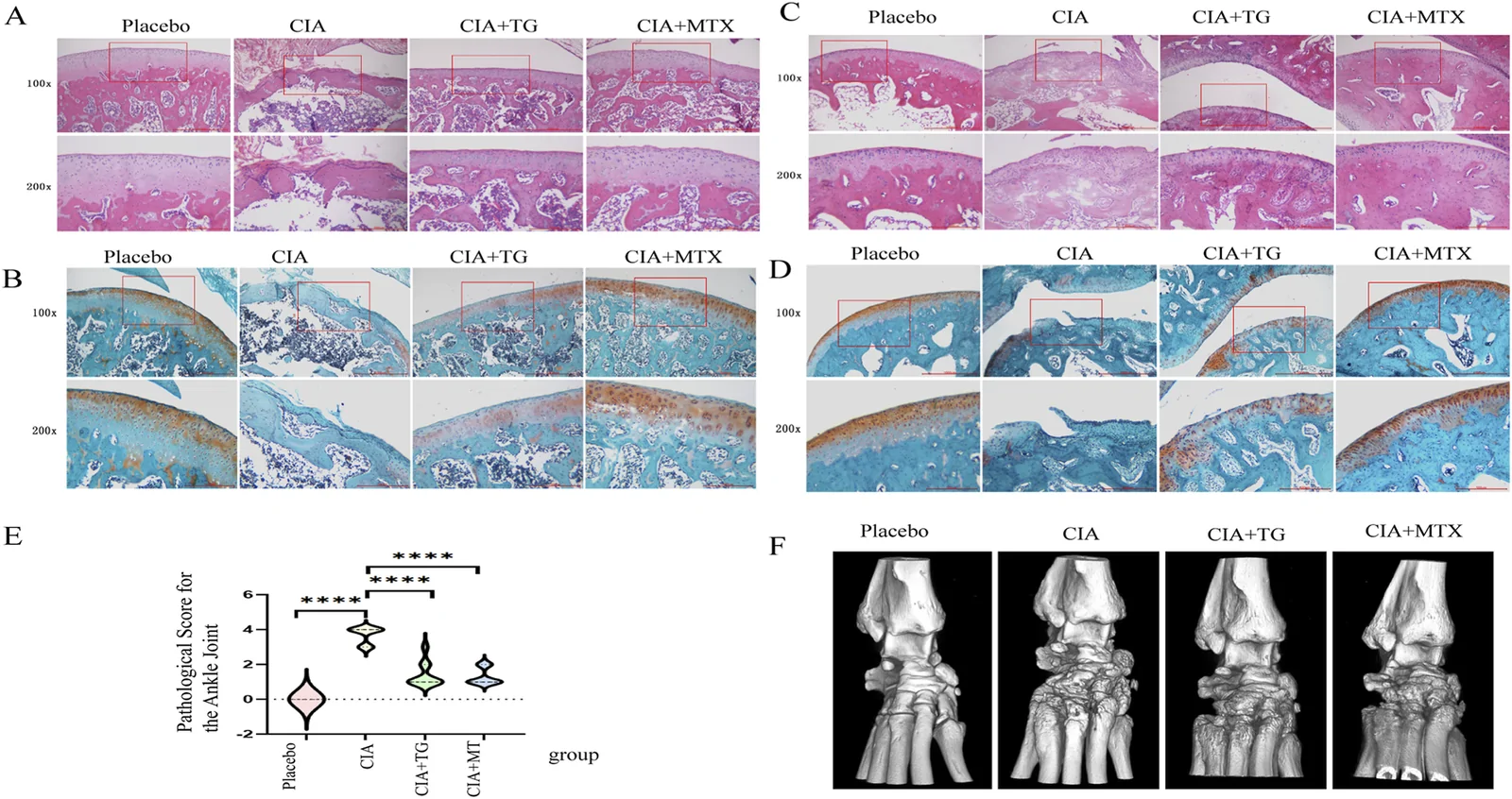
Results of HE staining, Safranin O-Fast Green staining, and micro-CT of the right knee and ankle joints in different rat groups after treatment. **(A)** HE staining results of the right knee joint. **(B)** Safranin O-Fast Green staining results of the right knee joint. **(C)** HE staining results of the right ankle joint. **(D)** Safranin O-Fast Green staining results of the right ankle joint. Magnification: ×100 and 200×; scale bars: 100 and 50 μm. **(E)** Pathological scores of the ankle joints. **(F)** Micro-CT related results of the right ankle joints in the four rat groups after treatment.

In the placebo group, HE staining showed intact cartilage and smooth surfaces, while the model group displayed severe structural destruction, bone damage, fibrosis, and synovial proliferation. TG and MTX treatments preserved cartilage integrity, with only mild synovial thickening (Figures 1A,C,E). Similarly, Safranin O-Fast Green staining revealed severe cartilage loss and glycosaminoglycan depletion in the model group relative to the well-preserved placebo group. The TG and MTX groups maintained structural integrity despite some glycosaminoglycan loss (Figures 1B,D). Micro-CT imaging indicated that the model group exhibited severe osteoporosis, joint space narrowing, and rough articular surfaces. Both the TG and MTX groups significantly attenuated these bone changes and structural disruptions, showing markedly less severity than the model group (Figure 1F; Table 1).

**TABLE 1:** Results of Micro-CT related parameters in rat ankle joints across groups (x‐ ± S).

Parameter	Placebo group	Model group	MTX group	TG group
N	10	10	10	10
BV/TV (%)	0.391 ± 0.020	0.262 ± 0.089***	0.453 ± 0.016**,###	0.468 ± 0.044***,###
BS/BV (1/mm)	40.289 ± 1.550	55.445 ± 12.170*	37.608 ± 2.414##	34.554 ± 3.088***,##
Tb.Th (mm)	0.051 ± 0.004	0.037 ± 0.010**	0.058 ± 0.007###	0.056 ± 0.004###
Tb.N (1/mm)	7.854 ± 0.320	6.497 ± 0.737***	8.467 ± 0.503**,###	8.290 ± 0.287*,###
Tb.Sp (mm)	0.077 ± 0.005	0.112 ± 0.036	0.067 ± 0.005**,#	0.054 ± 0.004***,##
TBPf (1/mm)	14.270 ± 1.922	14.518 ± 0.886	10.170 ± 2.408**,##	5.901 ± 0.615***,##
BMD (mg/cc)	2,359.837 ± 39.702	2,322.675 ± 41.188*	2,352.059 ± 23.879	2,321.744 ± 38.476
BS (mm^2^)	84.734 ± 17.786	166.713 ± 12.610*	149.939 ± 66.540	109.866 ± 10.272*,#
BV (mm^3^)	2.109 ± 0.473	3.159 ± 0.815*	3.962 ± 1.686*	3.147 ± 0.428*
BS/TV (1/mm)	15.749 ± 0.689	13.725 ± 1.859*	17.067 ± 1.500#	15.690 ± 1.993#

n = 10. Data are presented as Mean ± SEM.

* *P* < 0.05.

** *P* < 0.01.

*** *P* < 0.001 vs. the placebo group.

# *P* < 0.05.

## *P* < 0.01.

### *P* < 0.001 vs. the model group.

##### 3.1.1.1 Expression of JAK1, p-JAK1, STAT1, and p-STAT1 in ankle joints post-treatment

**FIGURE 2:**
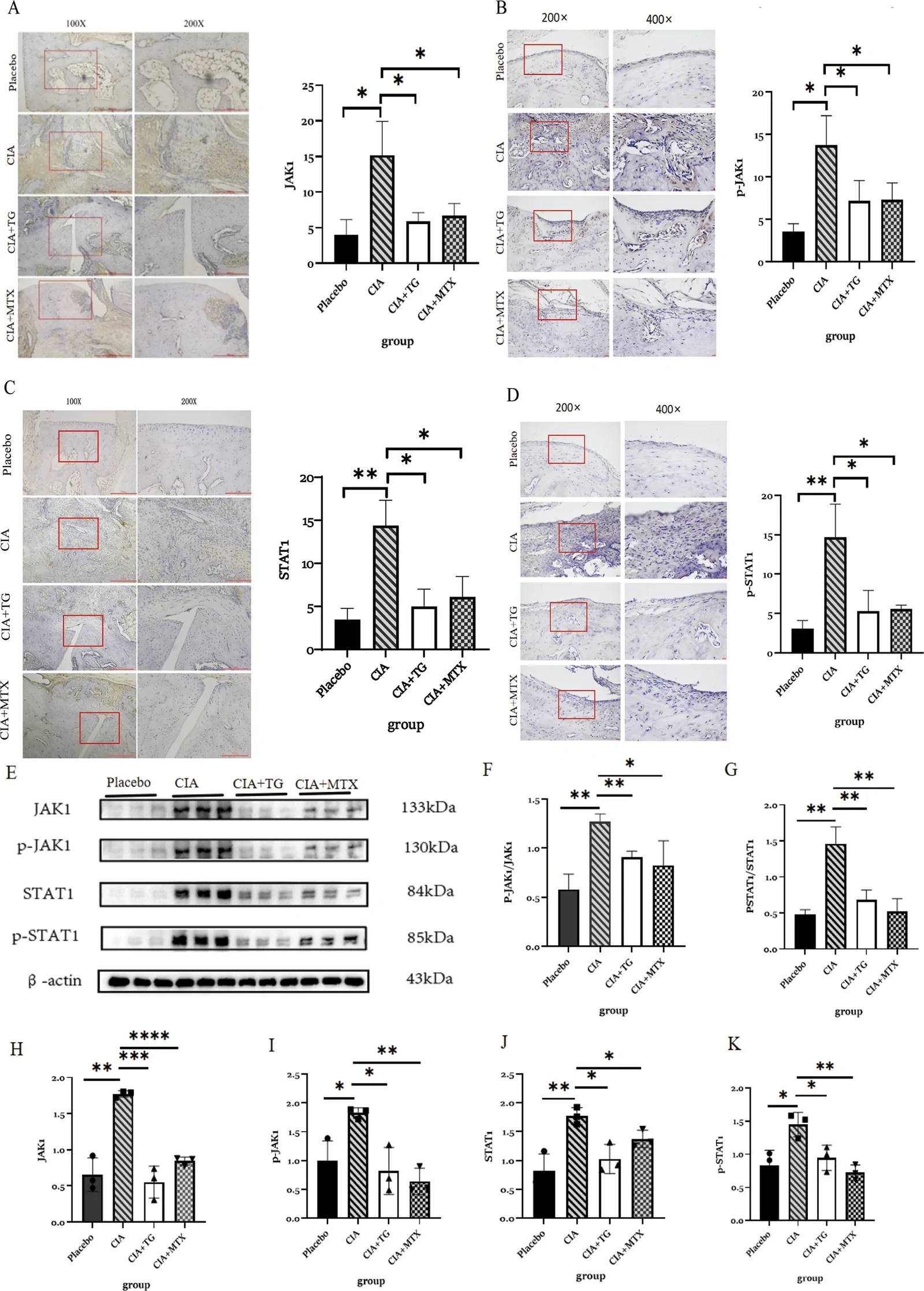
Protein expression of JAK1, STAT1, p-JAK1, and p-STAT1 in the ankle joints of rats. **(A)** Immunohistochemical staining and statistical analysis of JAK1 expression in the synovial tissue of the left knee joint in the CIA animal model. **(B)** Immunohistochemical staining and statistical analysis of p-JAK1 expression in the synovial tissue of the left knee joint in the CIA animal model. **(C)** Immunohistochemical staining and statistical analysis of STAT1 expression in the synovial tissue of the left knee joint in the CIA animal model. **(D)** Immunohistochemical staining and statistical analysis of p-STAT1 expression in the synovial tissue of the left knee joint in the CIA animal model. **(E)** WB analysis of JAK1, STAT1, p-JAK1, and p-STAT1 in the synovial tissue of the left knee joint in the CIA animal model. **(F)** WB analysis of p-JAK1/JAK1 in the synovial tissue of the left knee joint in the CIA animal model. **(G)** Statistical results of the p-STAT1/STAT1 ratio from WB analysis of the synovial tissue of the left knee joint in the CIA animal model. **(H)** Statistical results of JAK1 expression from WB analysis of the synovial tissue of the left knee joint in the CIA animal model. **(I)** Statistical results of p-JAK1 expression from WB analysis of the synovial tissue of the left knee joint in the CIA animal model. **(J)** Statistical results of STAT1 expression from WB analysis of the synovial tissue of the left knee joint in the CIA animal model. **(K)** Statistical results of p-STAT1 expression from WB analysis of the synovial tissue of the left knee joint in the CIA animal model. n = 3. Data are presented as mean ± SEM. *P < 0.05, **P < 0.01, ***P < 0.001, ****P < 0.0001.

IHC staining showed that JAK1, p-JAK1, STAT1, and p-STAT1 were localized in the synovium and cartilage layers. Their expression was significantly higher in the model group than in the placebo group (*P* < 0.05). Both TG and MTX treatments significantly suppressed this increase (*P* < 0.05 vs. model), restoring expression to levels similar to those in the placebo group (Figures 2A–D). The result of the TG group and MTX group were similar (*P* > 0.05).

Consistently, WB analysis of synovial tissues revealed that protein levels of JAK1, p-JAK1, STAT1, and p-STAT1 were significantly elevated in the model group (*P* < 0.05 vs. placebo) and were markedly reduced by both TG and MTX treatments (*P* < 0.05 vs. model). The inhibitory effect of TG was comparable to that of MTX (Figures 2E–K), indicating that TG effectively inhibits JAK/STAT pathway activation in CIA rat.

##### 3.1.1.2 Expression of JAK2, p-JAK2, STAT3, and p-STAT3 in ankle joints post-treatment

**FIGURE 3:**
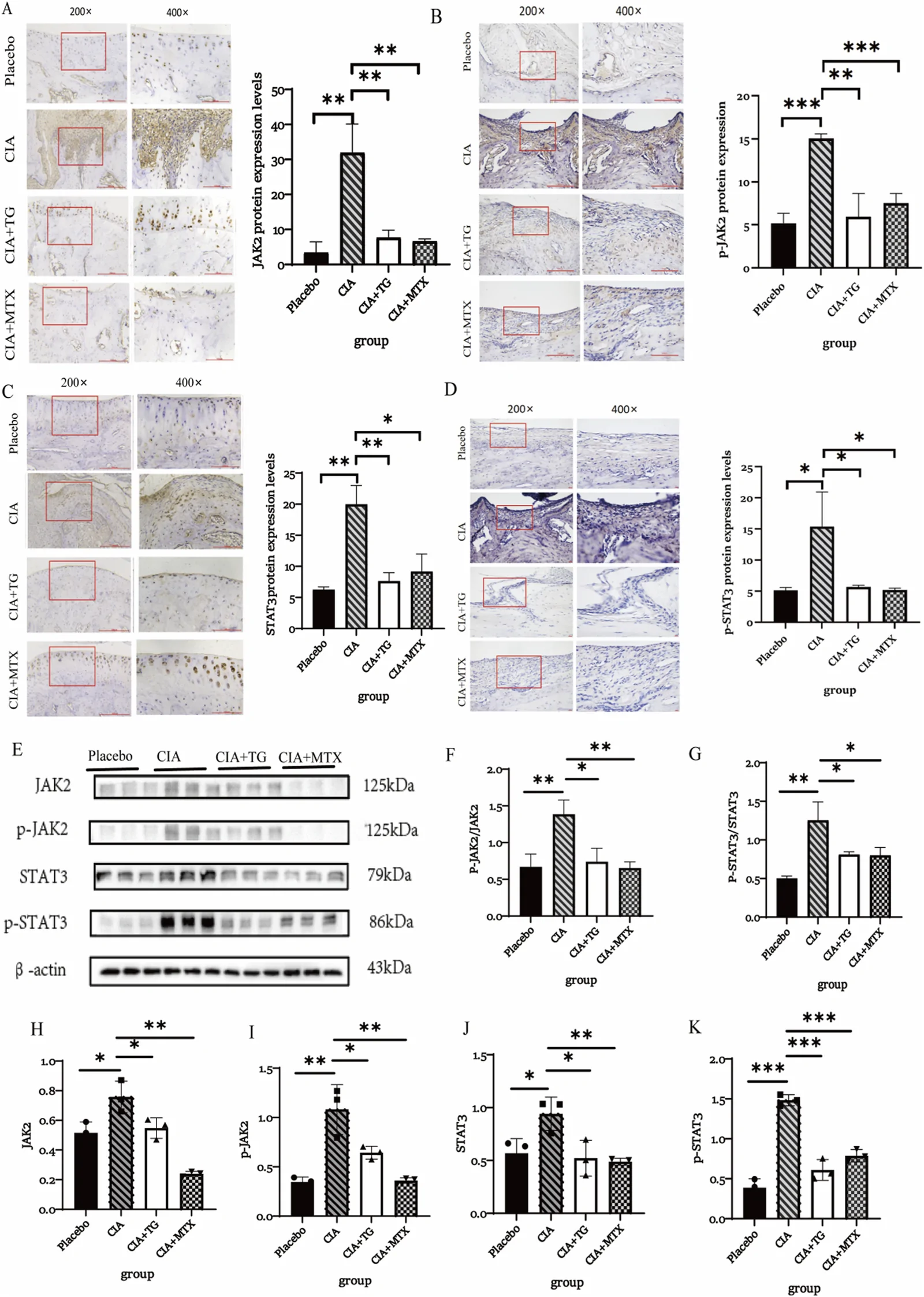
Expression of JAK2, p-JAK2, STAT3, and p-STAT3 in the rat ankle joint and synovial tissue. **(A)** Immunohistochemical staining and statistical results for JAK2 expression in the synovial tissue of the left knee joint from the CIA animal model. **(B)** Immunohistochemical staining and statistical results for p-JAK2 expression in the synovial tissue of the left knee joint from the CIA animal model. **(C)** Immunohistochemical staining and statistical results for STAT3 expression in the synovial tissue of the left knee joint from the CIA animal model. **(D)** Immunohistochemical staining and statistical results for p-STAT3 expression in the synovial tissue of the left knee joint from the CIA animal model. **(E)** WB analysis of JAK2, STAT3, p-JAK2, and p-STAT3 in the synovial tissue of the left knee joint from the CIA animal model. **(F)** WB analysis of p-JAK2/JAK2, STAT3, p-JAK2, and p-STAT3 in the synovial tissue of the left knee joint from the CIA animal model. **(G)** Statistical results of the p-STAT3/STAT3 ratio from WB analysis of the left knee synovial tissue in the CIA animal model. **(H)** Statistical results of JAK2 expression from WB analysis of the left knee synovial tissue in the CIA animal model. **(I)** Statistical results of p-JAK2 expression from WB analysis of the left knee synovial tissue in the CIA animal model. **(J)** Statistical results of STAT3 expression from WB analysis of the left knee synovial tissue in the CIA animal model. **(K)** Statistical results of p-STAT3 expression from WB analysis of the left knee synovial tissue in the CIA animal model. n = 3. Data are presented as mean ± SEM. *P < 0.05, **P < 0.01, ***P < 0.001.

IHC staining revealed that JAK2, p-JAK2, STAT3, and p-STAT3 were localized within the synovium and cartilage layers. Their expression was significantly higher in the model group than in the placebo group (*P* < 0.05). Both TG and MTX treatments markedly reduced this upregulation (*P* < 0.05 vs. model), restoring expression to levels comparable with those of the placebo group (Figures 3A–D). The effects of TG and MTX were similar (*P* > 0.05).

Consistently, WB analysis of synovial tissue showed that the protein levels of JAK2, p-JAK2, STAT3, and p-STAT3 were significantly elevated in the model group (*P* < 0.05 vs. placebo) and were significantly suppressed by both TG and MTX treatments (*P* < 0.05 vs. model). The inhibitory effect of TG was similar to that of MTX (Figures 3E–K), indicating that TG effectively inhibits activation of the JAK2/STAT3 signaling pathway in the joints of CIA rats.

##### 3.1.1.3 Expression of IGF1 in the ankle joint synovium of CIA model rats after treatment

**FIGURE 4:**
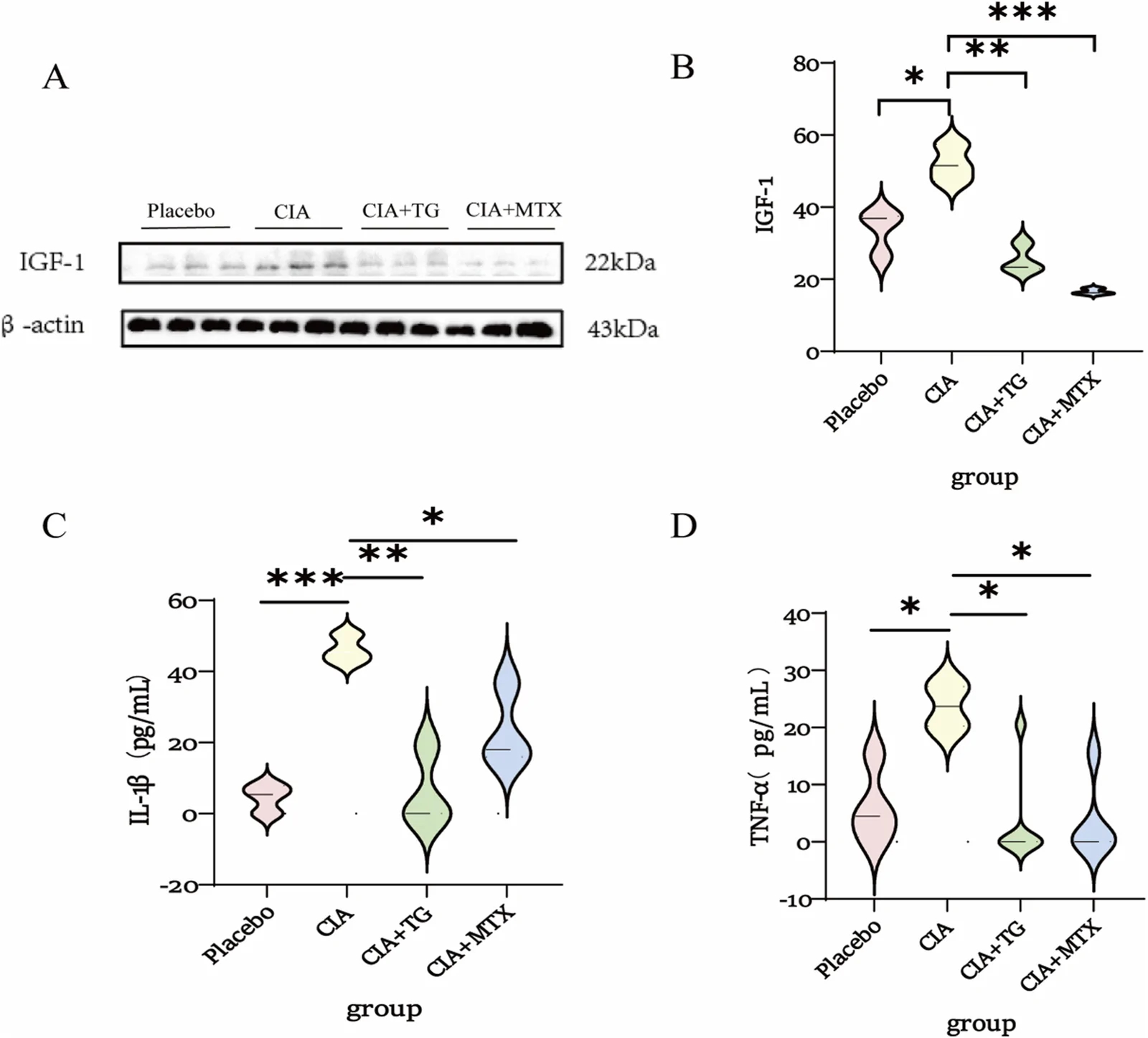
Protein expression of IGF1 in the synovial tissue of the left knee joint and serum levels of IL-1β and TNF-α in the CIA animal model. **(A)** Immunohistochemical staining for IGF1 expression in the synovial tissue of the left knee joint of the CIA animal model. **(B)** Statistical results of the immunohistochemical staining for IGF1 in the synovial tissue of the left knee joint of the CIA animal model. **(C)** Serum levels of IL-1β in the CIA animal model. **(D)** Serum levels of TNF-α in the CIA animal model. n = 3. Data are presented as Mean ± SEM. *P < 0.05, **P < 0.01, ***P < 0.001.

WB analysis results showed that compared with the placebo group, the protein expression of IGF1 in the knee joint synovium was significantly increased in the model group (P < 0.05). The protein expression in the TG group and the MTX group was significantly decreased compared to the model group (P < 0.01; P < 0.001, respectively). The expression levels in the MTX group and the TG group were similar (P > 0.05), indicating that TG can inhibit the expression of IGF1 in the ankle joint synovium of rats, with an effect comparable to that of MTX. Details are shown in Figures 4A,B.

#### 3.1.2 TG inhibits the expression of inflammatory factors IL-1β and TNF-α in the serum of CIA model rats

ELISA analysis of rat serum showed that IL-1β and TNF-α levels were significantly elevated in the model group compared to the placebo group (*P* < 0.05). Both TG and MTX treatments markedly reduced serum levels of these inflammatory cytokines (*P* < 0.05 vs. model), with no significant difference observed between the two treatment groups (*P* > 0.05), as shown in Figures 4C,D.

### 3.2 Cell experiment results

#### 3.2.1 Effects of TG on FLS

##### 3.2.1.1 Effect on FLS proliferation

CCK-8 assays were used to evaluate the cytotoxicity and anti-proliferative effects of TG-containing serum on FLS. Results showed that 15% drug-containing serum did not cause significant cytotoxicity. Compared with the placebo group, TG-containing serum effectively suppressed FLS proliferation, with the strongest effect observed at 24 h of treatment (Supplementary Figure 7). Under LPS-induced inflammatory conditions, treatment with 10% TG-containing serum for 24 h exhibited no cytotoxicity and achieved the most favorable inhibitory effect (Supplementary Table 3). Therefore, 10% TG-containing serum applied for 24 h was chosen as the standard intervention for subsequent.

##### 3.2.1.2 Effect of TG on FLS migration

Scratch wound healing assays showed that LPS stimulation significantly promoted FLS migration in the model group. Both TG and MTX treatments markedly reduced this migratory capacity, with no significant difference in efficacy between the two groups (Supplementary Figure 8).

##### 3.2.1.3 TG inhibits JAK-STAT pathway-related proteins in FLS

**FIGURE 5:**
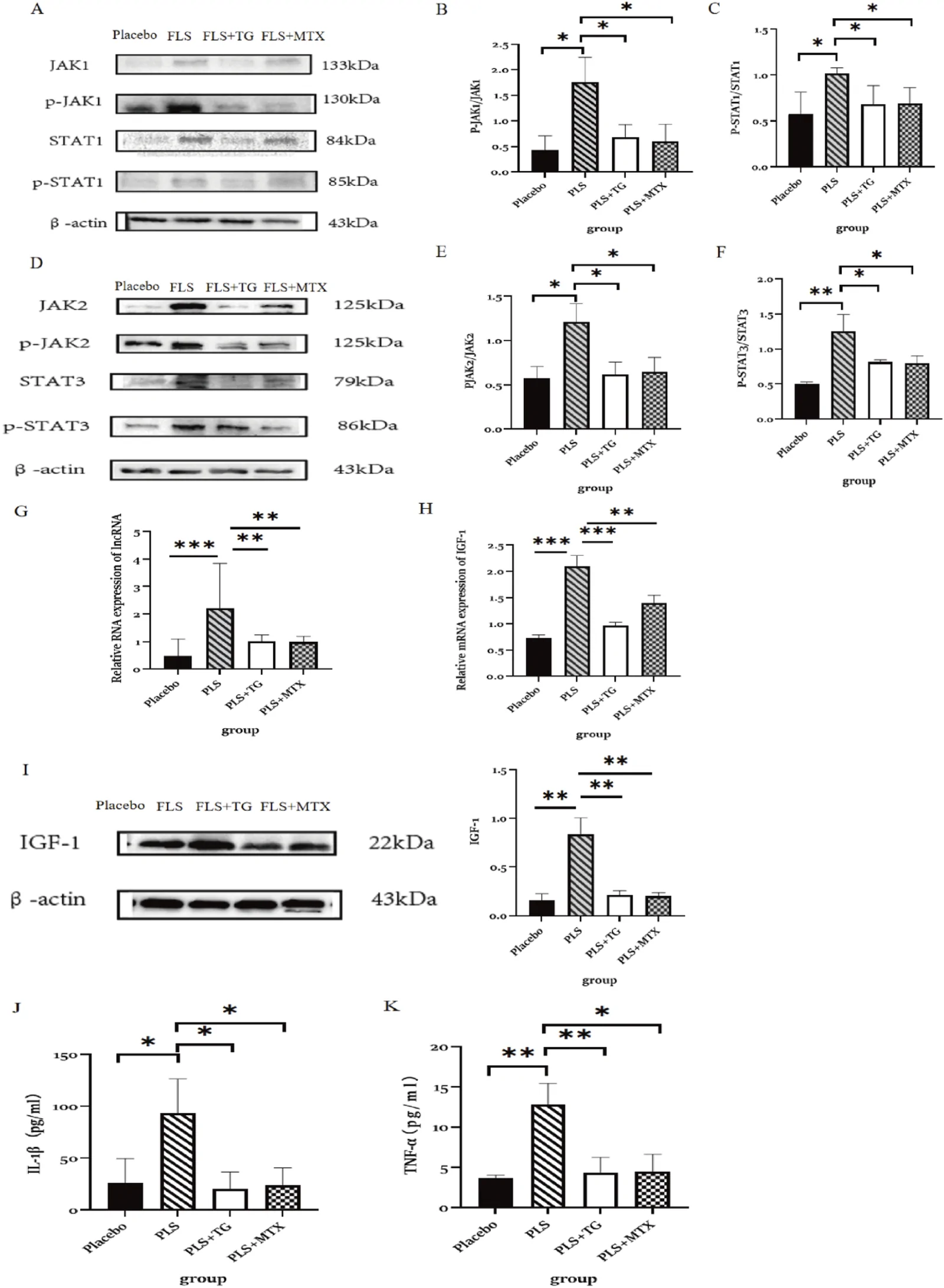
Expression of JAK-STAT pathway metabolites, Lnc-ENST00000602558, and IGF1 in FLS from different groups after intervention. **(A)** WB analysis of JAK1, STAT1, p-JAK1, and p-STAT1 in FLS. **(B)** Statistical results of p-JAK1/JAK1 ratio from WB analysis in FLS. **(C)** Statistical results of p-STAT1/STAT1 ratio from WB analysis in FLS. **(D)** WB analysis of JAK2, STAT3, p-JAK2, and p-STAT3 in FLS. **(E)** Statistical results of p-JAK2/JAK2 ratio from WB analysis in FLS. **(F)** Statistical results of p-STAT3/STAT3 ratio from WB analysis in FLS. **(G)** Expression of Lnc-ENST00000602558 in FLS after intervention with LPS and drug-containing serum. **(H)** Expression of IGF1 mRNA in FLS after intervention with LPS and drug-containing serum. **(I)** WB analysis of IGF1 protein in FLS. **(J)** Statistical results of IGF1 protein expression from WB analysis in FLS. **(J)** Expression of IL-1β in cell culture supernatants; **(K)** Expression of TNF-α in cell culture supernatants. n = 3. Data are presented as Mean ± SEM. *P < 0.05, **P < 0.01. n = 3. Data are presented as Mean ± SEM. *P < 0.05, **P < 0.01, ***P < 0.001.

WB analysis revealed that protein levels of JAK1, p-JAK1, STAT1, p-STAT1, JAK2, p-JAK2, STAT3, and p-STAT3 were significantly upregulated in the model group (*P* < 0.05 vs. placebo). Both TG and MTX treatments significantly suppressed this upregulation (*P* < 0.05 vs. model), restoring expression levels to near those of the placebo group. The inhibitory effect of TG was comparable to that of MTX (Figures 5A–F).

##### 3.2.1.4 TG inhibits Lnc-ENST00000602558 and IGF1 expression in FLS

RT-PCR and WB analyses demonstrated that expression of Lnc-ENST00000602558 and IGF1 (mRNA and protein) was significantly elevated in the model group (*P* < 0.05 vs. placebo). Both TG and MTX treatments markedly reduced their expression (*P* < 0.05 vs. model), bringing levels close to those in the placebo group. TG treatment resulted in a particularly pronounced reduction in IGF1 protein levels (Figures 5G–I).

##### 3.2.1.5 TG inhibits TNF-α and IL-1β expression in FLS

ELISA results showed that the secretion of TNF-α and IL-1β was markedly increased in the model group (*P* < 0.05 vs. placebo). Both TG and MTX treatment significantly reduced the levels of these inflammatory cytokines (*P* < 0.05 vs. model), bringing them back to levels similar to those in the placebo group. The inhibitory effect of TG was comparable to that of MTX (*P* > 0.05), as illustrated in Figures 5J,K.

#### 3.2.2 Dual-luciferase reporter assay

The results are shown in Supplementary Figure 9. The luciferase activity in Group 2 and Group 3 was significantly higher than that in Group 1 (*P* < 0.01; *P* < 0.0001), indicating successful construction of the IGF1 plasmid and the ENST00000602558 plasmid, which promoted luciferase expression. The luciferase activity in Group 4 was significantly higher than that in Group 2 and Group 3 (*P* < 0.0001), suggesting that the mutant ENST00000602558 sequence can specifically bind to IGF1.

#### 3.2.3 Effects of TG on FLS following LV-shlnc2558 infection

##### 3.2.3.1 Effects of TG on FLS with low expression of Lnc-ENST00000602558

To verify the impact of Lnc-ENST00000602558 on the migration and invasion of FLS, FLS were first infected with LV-shlnc2558 to inhibit Lnc-ENST00000602558. The infection results demonstrated a significant decrease in the gene level of Lnc-ENST00000602558 in FLS post-infection (*P* < 0.05), indicating successful Lnc-ENST00000602558 interference (Supplementary Figure 10).

**FIGURE 6:**
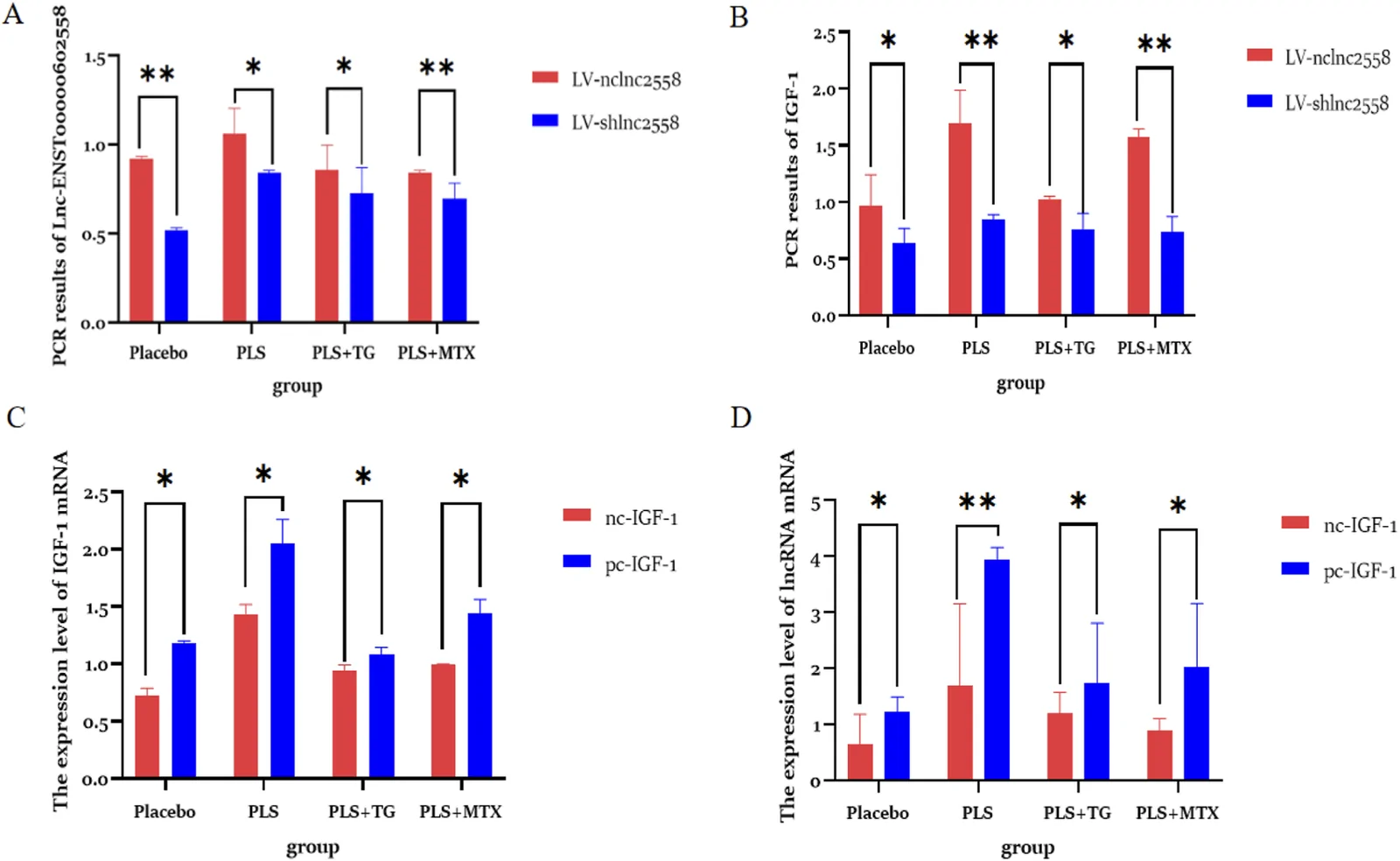
Expression of Lnc-ENST00000602558 and IGF1 genes in FLS cells following drug intervention after LV-shlnc2558 virus and LV-pcIGF1 virus infection. **(A)** mRNA expression of Lnc-ENST00000602558; **(B)** mRNA expression of IGF1; **(C)** Expression level of the Lnc-ENST00000602558 gene; **(D)** Expression level of IGF1 mRNA. n = 3. Data are presented as Mean ± SEM. *P < 0.05, **P < 0.01.

Subsequently, FLS infected with LV-shlnc2558 were treated with serum containing TG or MTX. It was found that the gene expression level of Lnc-ENST00000602558 in LV-shlnc2558-infected FLS was lower compared to that in the corresponding LV-nclnc2558-infected placebo group (*P* < 0.05). Following treatment with serum containing TG or MTX, the gene level of Lnc-ENST00000602558 in LV-shlnc2558-infected FLS showed a slight decrease; however, the difference compared to the PLS group was not statistically significant (*P* > 0.05). Specific data are presented in Figures 6A,B.

##### 3.2.3.2 Effect of TG on the migration of LV-shlnc2558-infected FLS

Scratch wound assays showed that knockdown of Lnc-ENST00000602558 via LV-shlnc2558 infection significantly reduced LPS-induced FLS migration (*P* < 0.05 vs. control). Although TG or MTX treatment further decreased the scratch wound area in the knockdown cells, this added suppression was not statistically significant compared to the untreated knockdown group (*P* > 0.05) (Supplementary Figure 11). These results indicate that inhibiting Lnc-ENST00000602558 inherently suppresses FLS migration, thereby diminishing the therapeutic effect.

##### 3.2.3.3 Effects of TG on the JAK-STAT signaling pathway and IGF1 protein expression in FLS infected with LV-shlnc2558

To verify the effects of TG on the expression of JAK-1, JAK-2, STAT-1, and STAT-3 proteins in FLS, FLS were infected with LV-shlnc2558, stimulated with LPS, and then cultured with drug-containing serum for 24 h. Subsequently, proteins were extracted to detect the expression of JAK-STAT pathway proteins and IGF1 protein in the cells.

###### 3.2.3.3.1 Effects of TG on JAK-STAT pathway proteins in LV-shlnc2558-infected FLS

**FIGURE 7:**
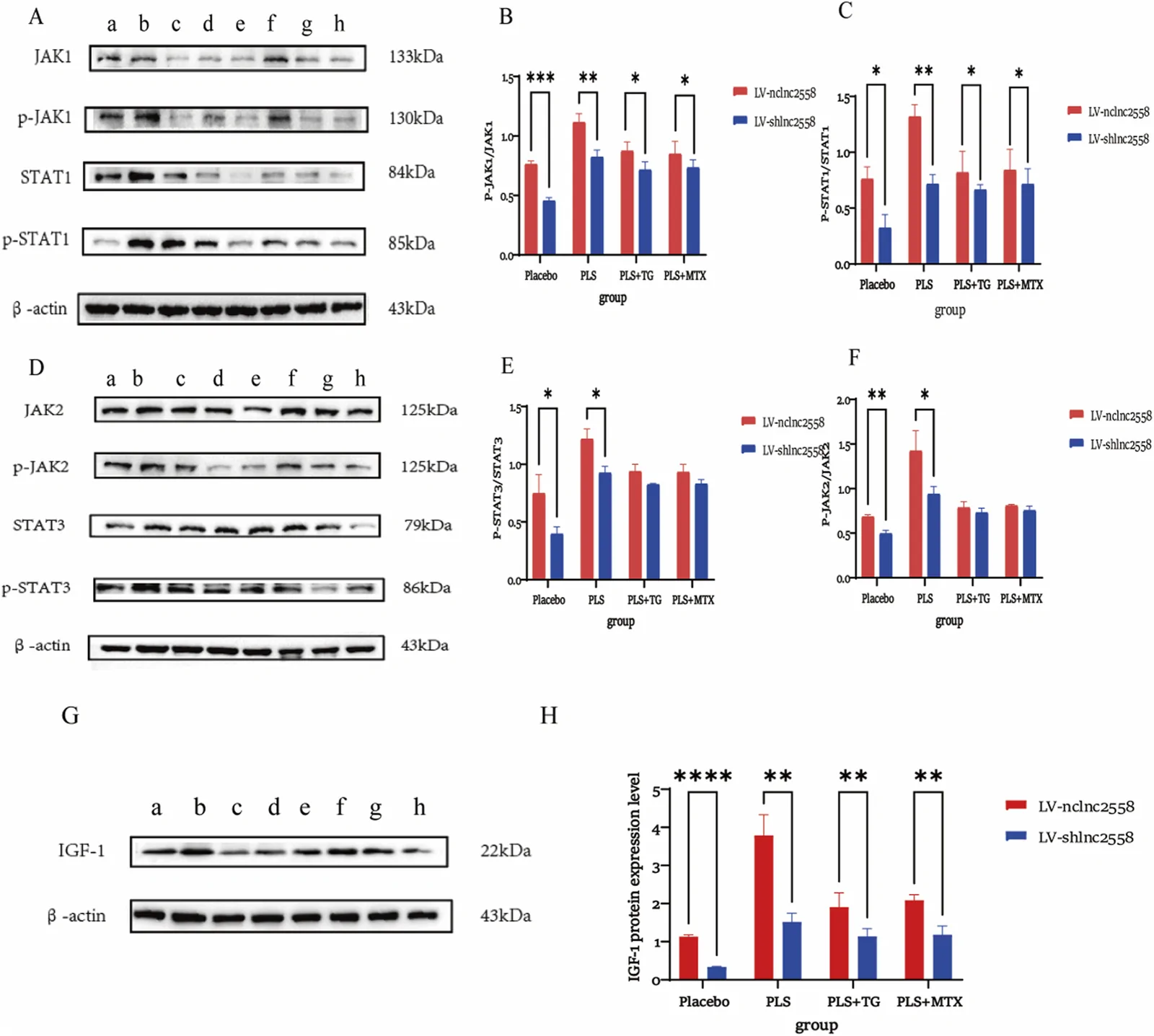
Expression of JAK-STAT pathway and IGF1-related proteins in FLS infected with LV-shlnc2558. **(A)** WB analysis of JAK1, p-JAK1, STAT1, and p-STAT1 in FLS. **(B)** Statistical results of p-JAK1/JAK1 protein expression in FLS from WB analysis. **(C)** Statistical results of p-STAT1/STAT1 protein expression in FLS from WB analysis. **(D)** WB analysis of JAK2, p-JAK2, STAT3, and p-STAT3 in FLS. **(E)** Statistical results of p-JAK2/JAK2 protein expression in FLS from WB analysis. **(F)** Statistical results of p-STAT3/STAT3 protein expression in FLS from WB analysis. **(G)** WB analysis of IGF1 in FLS infected with LV-shlnc2558. **(H)** Statistical results of IGF1 protein expression in FLS infected with LV-shlnc2558 from WB analysis. a: LV-nclnc2558 + Placebo group, b: LV-nclnc2558 + LPS + Placebo group, c: LV-nclnc2558 + LPS + TG group, d: LV-nclnc2558 + LPS + MTX group, e: LV-shlnc2558 + Placebo group, f: LV-shlnc2558 + LPS + Placebo group, g: LV-shlnc2558 + LPS + TG group, **(H)** LV-shlnc2558 + LPS + MTX group. n = 3. Data are presented as mean ± SEM. *P < 0.05, **P < 0.01, ***P < 0.001, ****P < 0.0001.

Protein expression analysis indicated that knockdown of Lnc-ENST00000602558 via LV-shlnc2558 infection markedly reduced basal levels of JAK1, p-JAK1, STAT1, p-STAT1, JAK2, p-JAK2, STAT3, and p-STAT3 compared with the blank control group (*P* < 0.05). In these knockdown cells, LPS stimulation significantly upregulated the expression of these JAK-STAT proteins (*P* < 0.05 vs. blank). Although TG and MTX treatments attenuated the LPS-induced upregulation, the reductions did not reach statistical significance (*P* > 0.05) (Figures 7A–F).

###### 3.2.3.3.2 Effects of TG on IGF1 protein expression in LV-shlnc2558-infected FLS

IGF1 protein expression was similarly lower in LV-shlnc2558-infected cells than in the blank control group (*P* < 0.05). In these knockdown cells, LPS stimulation significantly decreased IGF1 levels (*P* < 0.05 vs. blank). Subsequent TG and MTX treatments further reduced IGF1 expression slightly, but the changes were not statistically significant (*P* > 0.05) (Figures 7G,H).

#### 3.2.4 Effect of TG on IGF1-Overexpressing FLS

To investigate the impact of IGF1 on the migration and invasion of FLS, we first infected FLS with the lentiviral vector LV-pcIGF1 to induce IGF1 overexpression. The infection results demonstrated that both the mRNA and protein levels of IGF1 in FLS were significantly increased post-infection (*P* < 0.0001), confirming successful IGF1 overexpression. For details, please refer to Supplementary Figures 12, 13.

##### 3.2.4.1 Effect of TG on Lnc-ENST00000602558 and IGF1 expression in IGF1-Overexpressing FLS

In FLS infected with LV-pcIGF1, the expression levels of both Lnc-ENST00000602558 and IGF1 were significantly elevated compared to the LV-ncIGF1 control group (*P* < 0.05). Treatment with TG- or MTX-containing serum significantly reduced the expression of both genes (*P* < 0.05 vs. FLS group) (Figures 6C,D).

##### 3.2.4.2 Effect of TG on the migration of LV-pcIGF1-infected FLS

Scratch wound assays demonstrated that IGF1 overexpression (LV-pcIGF1 infection) significantly promoted FLS migration compared to the LV-ncIGF1 control (*P* < 0.05). In LV-pcIGF1-infected FLS, both TG and MTX treatments significantly attenuated migration compared to the PLS group (*P* < 0.05) (Supplementary Figure 14).

##### 3.2.4.3 Effects of TG on the JAK-STAT pathway and IGF1 protein expression in LV-pcIGF1-infected FLS

**FIGURE 8:**
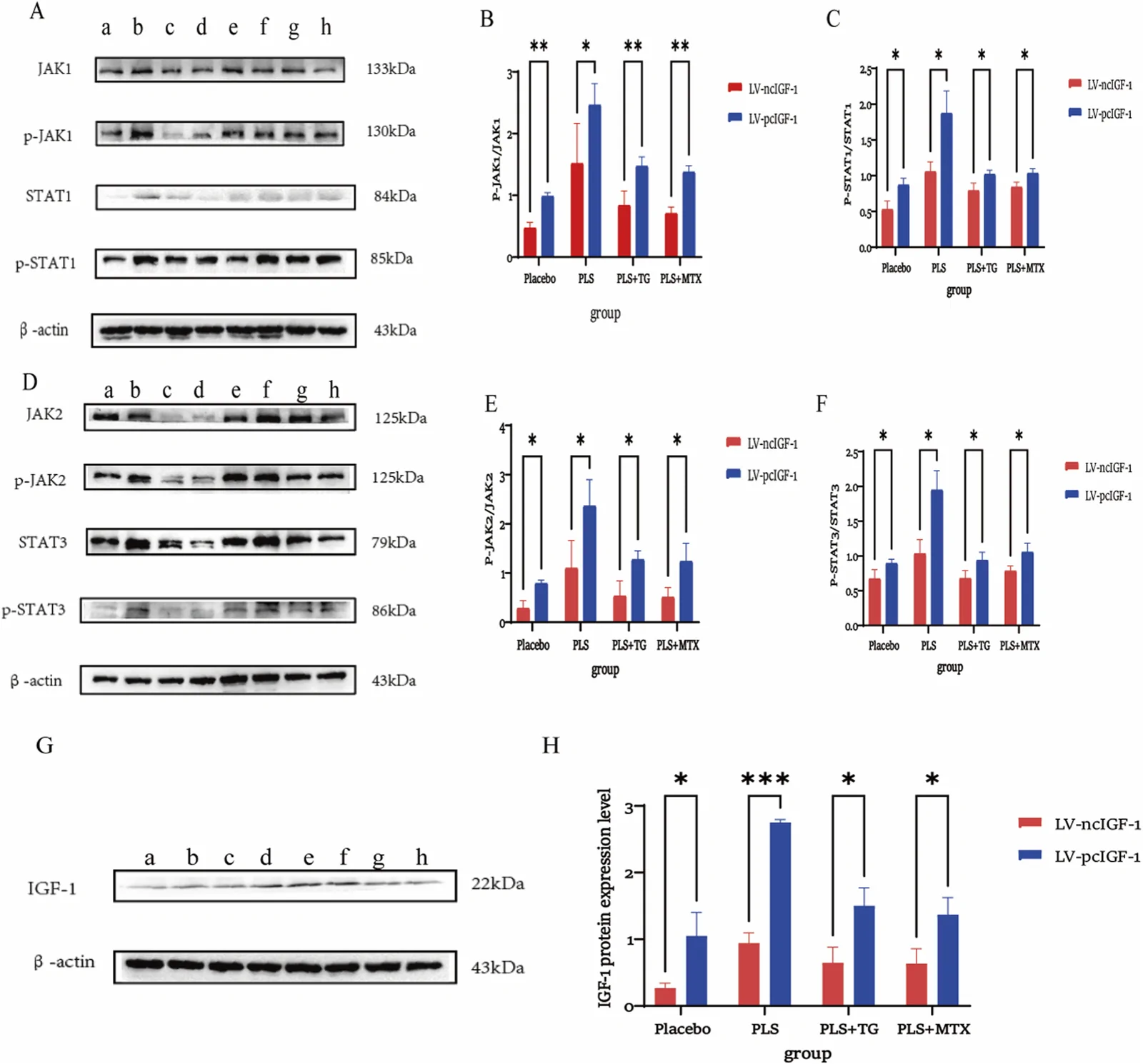
Expression of JAK-STAT signaling pathway proteins in FLS following LV-pcIGF1 infection. **(A)** WB analysis of JAK1, p-JAK1, STAT1, and p-STAT1 in FLS after LV-pcIGF1 infection. **(B)** Statistical results of the p-JAK1/JAK1 ratio from WB analysis in FLS after LV-pcIGF1 infection. **(C)** Statistical results of the p-STAT1/STAT1 ratio from WB analysis in FLS after LV-pcIGF1 infection. **(D)** WB analysis of JAK2, p-JAK2, STAT3, and p-STAT3 in FLS after LV-pcIGF1 infection. **(E)** Statistical results of the p-JAK2/JAK2 ratio from WB analysis in FLS after LV-pcIGF1 infection. **(F)** Statistical results of the p-STAT3/STAT3 ratio from WB analysis in FLS after LV-pcIGF1 infection. **(G)** WB analysis of IGF1 in FLS after LV-pcIGF1 infection. **(H)** Statistical results of IGF1 expression from WB analysis in FLS after LV-pcIGF1 infection. Group designations:a: LV-ncIGF1 + Placebo group; b: LV-ncIGF1 + LPS group; c: LV-ncIGF1 + LPS + TG group; d: LV-ncIGF1 + LPS + MTX group; e: LV-pcIGF1 + Placebo group; f: LV-pcIGF1 + LPS group; g: LV-pcIGF1 + LPS + TG group; h: LV-pcIGF1 + LPS + MTX group. n = 3. Data are presented as mean ± SEM. *P < 0.05, **P < 0.01, ***P < 0.001.

WB analysis revealed that IGF1 overexpression (LV-pcIGF1 infection) significantly elevated the phosphorylation ratios of p-JAK1/JAK1, p-STAT1/STAT1, p-JAK2/JAK2, and p-STAT3/STAT3 compared to the LV-ncIGF1 control (*P* < 0.05). LPS stimulation further upregulated JAK-STAT pathway activity and IGF1 protein expression in LV-pcIGF1-infected cells (*P* < 0.05 vs. LV-pcIGF1 + Placebo). TG treatment significantly attenuated this LPS-induced upregulation of both JAK-STAT signaling and IGF1 (*P* < 0.05 vs. LV-pcIGF1 + LPS), with efficacy comparable to that of MTX (*P* > 0.05) (Figures 8A–H).

## 4 Discussion

The results of this study indicate that TG alleviates bone and cartilage destruction in the ankle and knee joints, improves trabecular bone function, and reduces serum levels of TNF-α and IL-1β in CIA model rats, with efficacy comparable to that of MTX. WB and IHC results showed that, relative to the placebo group, the expression levels of JAK1, STAT1, p-STAT1, JAK2, p-JAK2, STAT3, p-JAK1, p-STAT3, and IGF-1 in the synovial tissue of the model group were significantly elevated (P < 0.05). After 4 weeks of TG treatment, the protein levels of the JAK/STAT pathway and IGF-1 in the synovial tissue were significantly reduced, with some outcomes surpassing those in the MTX group. These findings suggest that TG may improve the inflammatory state of RA and alleviate bone and cartilage destruction by modulating the IGF1 and JAK/STAT pathways, thereby inhibiting the expression of serum inflammatory factors.

**FIGURE 9:**
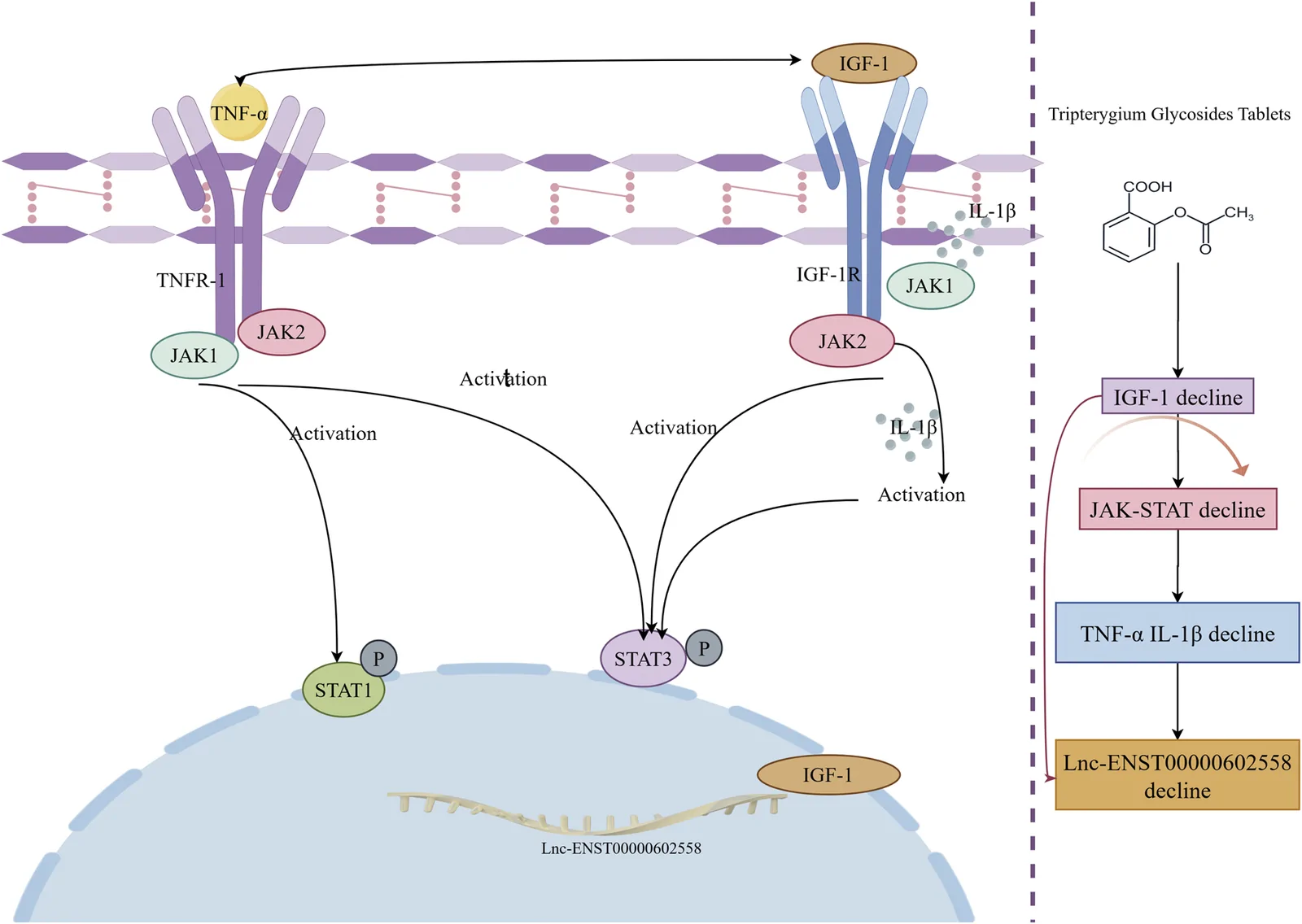
Mechanism of action of TG in the treatment of RA based on the Lnc-ENST00000602558/IGF1 signaling pathway.

The results of the dual-luciferase reporter assay showed that the mutant sequence of Lnc-ENST00000602558 could specifically bind to IGF1, and a positive regulatory relationship existed between them.The interaction among Lnc-ENST00000602558, IGF1, and the JAK/STAT pathway was further examined using lentiviral infection. Following Lnc-ENST00000602558 knockdown, the expression levels of IGF1 and JAK/STAT pathway-related targets were significantly reduced, and cell migration was slowed. Upon IGF1 overexpression, the protein levels of JAK/STAT pathway targets and the gene expression of Lnc-ENST00000602558 significantly increased, and cell migration accelerated. After TG treatment, the gene expression levels of Lnc-ENST00000602558 and IGF1 decreased. In FLS cells with low expression of Lnc-ENST00000602558, TG’s inhibitory effect on the JAK/STAT pathway was minimal, as shown in Figure 9.

The etiology of RA remains unclear but is generally considered to involve inflammation and immunity, with contributions from various immune cells and cytokines. Previous studies have shown that the endocrine system influences immune regulation through growth hormone activity, particularly IGF1. IGF1 is an anabolic growth hormone responsible for cell growth, differentiation, proliferation, and survival, and it also modulates immune-inflammatory responses. It serves as a key regulator of anabolic and catabolic pathways in skeletal muscle (Biagetti and Simó, 2021). IGF1 is closely associated with bone and cartilage turnover (Dixit et al., 2021). In certain rheumatic diseases, including osteoarthritis, diffuse idiopathic skeletal hyperostosis, and RA, abnormal serum and synovial fluid levels of IGF1 have been observed. However, the mechanisms through which IGF1 contributes to RA onset and progression remain poorly defined. Mechanistic studies in diseases such as diabetes and liver fibrosis, researchers have found that the JAK/STAT signaling pathway can regulate IGF1 expression (Sheng et al., 2017). Thus, IGF1 may be involved in the pathogenesis and progression of RA via the JAK/STAT signaling pathway.

The JAK/STAT signal transduction pathway is a common intracellular signaling pathway that directly links extracellular stimuli to gene expression. It participates in critical biological processes such as cell proliferation, differentiation, apoptosis, and immune regulation, and is closely associated with immune-inflammatory diseases (Xin et al., 2020). The JAK protein family comprises four members: JAK1, JAK2, JAK3, and TYK2 (Agashe et al., 2022). The STAT family includes seven proteins: STAT1, STAT2, STAT3, STAT4, STAT5A, STAT5B, and STAT6 (Erdogan et al., 2022). In particular, JAK1 and STAT1 are strongly linked to autoimmune diseases (Xue et al., 2023). FLS from patients with active RA also exhibit elevated STAT1 expression and activity. Mori T. et al. found that high levels of inflammatory cytokines in RA activate the IL-6-STAT3 cytokine loop, driving chronic persistent inflammation and joint destruction (Mori et al., 2011). Increased STAT3 activity in synovial CD4^+^ T cells in RA leads to a higher number of Th17 cells and a reduced number of Treg cells, which correlates closely with synovitis severity (Paradowska-Gorycka et al., 2020). Another study indicated that miR-17-5p inhibits the secretion of pro-inflammatory cytokines (such as IL-6, IL-1β) and binds to STAT3 and JAK1, thereby exerting anti-inflammatory and anti-erosive effect (Duan et al., 2022). Thus, STAT3 is also considered to plays an important role in RA disease progression.

JAK inhibitors have recently emerged as a new class of DMARDs that can reduce synovitis and systemic inflammation while improving function in RA patients. In 2019, EULAR positioned JAK inhibitors as equivalent to the use of biological DMARDs where conventional synthetic DMARDs prove ineffective. A large observational study on commonly used second-line RA drugs confirmed that JAK inhibitors outperform MTX (Zhou et al., 2018; Shu et al., 2020). These findings show that JAK inhibitors effectively alleviate symptoms and slow disease progression in RA, further underscoring the role of the JAK/STAT signaling pathway in RA pathogenesis and development.

In our previous study, we used gene expression microarrays to compare peripheral blood mononuclear cells from RA patients who responded to TG versus those who did not, identifying the Lnc-ENST00000602558/IGF1 axis as a key predictive marker. A partial least squares model based on the expression of this axis effectively predicted patient response to TG. Moreover, *in vitro* data showed that this axis mediates TG’s therapeutic efficacy by reducing TNF-α-induced IGF1 expression and inflammation in MH7A cells (Gao et al., 2024). No other reports on Lnc-ENST00000602558 in the treatment of RA with TG have been found in the domestic and international literature.

The JAK/STAT signaling pathway discovered in recent years, is an intracellular signal transduction cascade that can be activated by IGF1. Key cytokines involved in the pathogenesis and joint destruction of rheumatoid arthritis (RA), including IL-2, IL-6, IL-15, IL-17, IL-21, interferons, and granulocyte-macrophage colony-stimulating factor, all activate the JAK/STAT pathway through different routes, thus playing a significant role in RA pathogenesis (Sharma et al., 2022). Unlike biologics that target a single pro-inflammatory factor, drugs inhibiting any JAK family protein can exert therapeutic effects on RA. The regulation of the JAK/STAT signaling pathway by TG has been increasingly documented (Zhou et al., 2018; Liu et al., 2018; Huang et al., 2019).

A major innovation of this study is the elucidation of a novel mechanism by which TG treats RA, namely, suppression of the JAK/STAT signaling pathway through downregulation of Lnc-ENST00000602558 and IGF1. This finding extends beyond the traditional understanding of TG’s pharmacological actions by linking its therapeutic effects to the lncRNA regulatory network. As a key molecule identified in our research, Lnc-ENST00000602558 modulates IGF1 to influence the JAK/STAT pathway, providing new insights into RA pathogenesis. Compared with existing knowledge, our study not only confirms the importance of the Lnc-ENST00000602558/IGF1 axis and the JAK/STAT pathway in RA but, more importantly, clarifies the upstream regulatory mechanisms of this pathway, particularly the distinct role of Lnc-ENST00000602558. Thus, these findings deepen our understanding of the molecular mechanisms underlying RA and offer an experimental basis for developing lncRNA-based therapeutic strategies, with considerable potential for clinical translation.

This study has several limitations. First, the findings primarily reflect the overall effects of the commonly used clinical preparation TG, without analyzing its individual metabolites. Potential discrepancies may exist between studies of single active metabolites and those of composite formulations. Future research should employ fingerprint technology to further characterize the active metabolites and address this gap in the current data. Second, this study did not include a comprehensive assessment of TG toxicity during RA treatment. Subsequent studies should incorporate relevant analyses to address this issue. Next, the animal study did not include inhibition of Lnc-ENST00000602558, which constitutes a limitation of the present experiment. Future research will address this gap by conducting comprehensive *in vivo* investigations to supplement the current data. Finally, although the CIA animal model used here is widely adopted in RA research, its inherent differences from human disease may limit the clinical translatability of the findings, which require further validation in clinical trials.

## 5 Conclusion

In summary, our study demonstrates that TG effectively ameliorates joint pathology in CIA rats by suppressing the JAK/STAT pathway, thereby reducing synovial hyperplasia, bone and cartilage destruction, and systemic inflammation. Mechanistically, our *ex vivo* experiments revealed a novel role for the Lnc-ENST00000602558/IGF1 axis as a critical upstream activator of JAK/STAT signaling in FLS. A particularly notable finding is that the therapeutic efficacy of TG may be highly dependent on this specific lncRNA axis; knockdown of Lnc-ENST00000602558 abolished the ability of TG to inhibit the JAK/STAT pathway. These results not only uncover a new molecular mechanism underlying the pharmacological action of TG but also implicate the Lnc-ENST00000602558/IGF1 axis as a promising therapeutic target and potential biomarker. Accordingly, this work provides valuable theoretical support for the development of novel targeted interventions and personalized treatment.

## Data Availability

The raw data supporting the conclusions of this article will be made available by the authors, without undue reservation.
