# Supplementary material for: Exploring the mechanism of action of tripterygium glycosides tablets in the treatment of rheumatoid arthritis based on the Lnc-ENST00000602558/IGF1 signaling pathway
Source: Front Pharmacol. 2026 Aug 20;17:1864355. doi: 10.3389/fphar.2026.1864355 (PMC13538848; doi:10.3389/fphar.2026.1864355)
Supplement: Supplementary file 3 [file Table1.docx]

According to the Chinese Pharmacopoeia, triptolide and wilforlide A are designated as the primary marker compounds. These two compounds were detected in TG tablets by LC-MS analysis. Triptolide has a molecular formula of C₂₀H₂₄O₆ and a molecular weight of 360.4. Wilforlide A has a molecular formula of C₃₀H₄₆O₃ and a molecular weight of 454.68. Supplementary Figure 1 shows the selected ion chromatogram (SIC) of triptolide in the TG tablets; Supplementary Figure 3 shows the corresponding chromatogram for wilforlide A in the same tablets.Methodological validation indicated that triptolide exhibited good linearity across a concentration range of 625 to 10,000 ng/mL (R² = 0.9999). The content per tablet was determined to be 0.720 μg, consistent with the reported value of 0.8 μg in the Certificate of Analysis (COA), as shown in Supplementary Figure 2. Wilforlide A also showed good linearity over a concentration range of 1,250 to 20,000 ng/mL (R² = 0.99994). The average content of wilforlide A in the sample was found to be 22.24 μg, comparable to the reported value of 21.2 μg in the COA, as illustrated in Supplementary Figure 4. In addition, relevant tests were also conducted on another batch of the drugs, and the results for triptolide and wilforlide A were similar to those of this experiment.

Supplementary Figure 1 SIC of Triptolide

Supplementary Figure 2 Quantitative analysis result of Triptolide

Supplementary Figure 3 SIC of Wilforlide A

Supplementary Figure 4 Quantitative analysis result of Wilforlide A

| Supplementary Table 1. Experimental Group of the Dual-Luciferase Dual-Fluoresceinase Reporter assay | |
| --- | --- |
| Group Number | Corresponding Group Name |
| group 1 | IGF1 promoter-luc-NC（ CON663）+ENST00000602558-NC（CON520） |
| group 2 | IGF1 promoter-luc-NC（ CON663）+ENST00000602558 （PL-ENST00000602558(K24K0007)） |
| group 3 | IGF1 promoter-luc（PL-IGF1(K24K0006)）+ENST00000602558-NC（CON520） |
| group 4 | IGF1 promoter-luc（PL-IGF1(K24K0006)）+ENST00000602558（PL-ENST00000602558(K24K0007) ） |

Supplementary Table 2 Primer and reference sequences for lncRNA ENST00000602558 and IGF1

| Species | Primer Name | Sequence (5'→3') |
| --- | --- | --- |
| Human | IGF1 Forward Primer | GGCATCGTGGATGAGTGCTG |
| Human | IGF1 Reverse Primer | GTCTTGGGCATGTCGGTGTG |
| Human | GAPDH Forward Primer | GGAGCGAGATCCCTCCAAAAT |
| Human | GAPDH Reverse Primer | GGCTGTTGTCATACTTCTCATGG |
| Human | 18s Forward Primer | GCGGCGGAAAATAGCCTTTG |
| Human | 18s Reverse Primer | GATCACACGTTCCACCTCATC |
| Human | LncRNAForward Primer | GCAGTACGATCTAGTCCACTAAGG |
| Human | LncRNAReverse Primer | AGTATTACAGCGTGGTAGCTTGC |

**Identification of FLS**

In this section, primary fibroblast-like synoviocytes (FLS) from rheumatoid arthritis (RA) patients were isolated using enzymatic digestion. FLS from passages 3 to 8 were used for subsequent experiments. The morphology of the primary FLS is shown in Supplementary Figure 5, displaying a characteristic long spindle shape with an oval nucleus centrally located within the cell.

.

Supplementary Figure 5 Morphology of primary FLS

Note: Magnification 40×; scale bar = 100 μm.

The extracted cells were identified as FLS by flow cytometry through detection of CD68 and Vimentin expression. Flow cytometry results indicated that in passage 3 FLS, 0.2% of cells were positive for CD68, while 99.6% of cells were positive for Vimentin. This suggests a minimal presence of macrophage-like synoviocytes in passage 3 FLS, confirming their suitability for further experiments, as shown in Supplementary Figure 6.

Supplementary Figure 6 Flow cytometry identification results of FLS

Note: Panel A: Gating strategy for acquired cells; Panel B: Histogram peak for Vimentin single-staining of FLS; Panel C: Histogram peak for CD68 single-staining of FLS.

Supplementary Figure 7 Effects of Different Concentrations of Drug-Containing Serum and Incubation Time on the Proliferation of FLS Cells

Supplementary Figure 8 Migration of FLS Cells in Different Groups

Note: Panel A: Representative images of FLS cell migration in different groups; Panel B: Statistical results of FLS cell migration in different groups. n = 3. Data are presented as Mean ± SEM. *P < 0.05, **P < 0.001. Magnification: 40×. Scale bar: 100 μm.

| Supplementary Table 3 Effect of different concentrations of drug-containing serum on the proliferation of LPS-stimulated FLS cells after 24 hours of culture | | | |
| --- | --- | --- | --- |
| Concentration of Drug-containing Serum | OD Value (FLS Group) | OD Value (Control Group) | OD Value (TG Group) |
| 5% | 0.799 | 0.788 | 0.747 |
| 10% | 0.88 | 0.728 | 0.714 |
| 15% | 0.771 | 0.865 | 0.855 |

Supplementary Figure 9. Results of the Dual-Luciferase Reporter Assay

Note: Group 1: IGF1 promoter-luc-NC (CON663) + ENST00000602558-NC (CON520);

Group 2: IGF1 promoter-luc-NC (CON663) + ENST00000602558 (PL-ENST00000602558(K24K0007)); Group 3: IGF1 promoter-luc (PL-IGF1(K24K0006)) + ENST00000602558-NC (CON520); Group 4: IGF1 promoter-luc (PL-IGF1(K24K0006)) + ENST00000602558 (PL-ENST00000602558(K24K0007)). n = 3. Data are presented as Mean + SEM. Compared with Group 1, **P < 0.01; compared with Group 1, ****P < 0.0001; compared with Group 4, ####P < 0.0001.

Supplementary Figure 10 RT-PCR Results of FLS Cells Following Viral Infection

Note: A: Bright-field and fluorescence images of FLS cells after infection with LV-shlnc2558 virus. B: Expression level of Lnc-ENST00000602558 in FLS cells after infection with LV-shlnc2558 virus. n = 3.

Data are presented as mean ± SEM. **P < 0.01. Magnification: 40×; scale bar: 100 μm.

Supplementary Figure 11 Migration of FLS cells following drug intervention after LV-shlnc2558 virus infection.

Note: Panel A: Images showing FLS cell migration in different groups after LV-shlnc2558 infection. a: LV-nclnc2558 + Placebo group, b: LV-nclnc2558 + LPS group, c: LV-nclnc2558 + LPS + TG group, d: LV-nclnc2558 + LPS + MTX group, e: LV-shlnc2558 + Placebo group, f: LV-shlnc2558 + LPS group, g: LV-shlnc2558 + LPS + TG group, h: LV-shlnc2558 + LPS + MTX group. Panel B: Statistical results of FLS cell migration in different groups after LV-shlnc2558 infection. n = 3. Data are presented as Mean ± SEM. **P < 0.01, ***P < 0.001. Magnification: 40×; scale bar: 100 μm.

To verify the effect of IGF1 on FLS migration and invasion, FLS cells were first infected with the LV-pcIGF1 lentivirus to induce IGF1 overexpression. The infection results demonstrated that both the mRNA and protein levels of IGF1 in FLS cells were significantly increased post-infection (P < 0.0001), confirming successful IGF1 overexpression. Details are shown in Figure 8 and Supplementary Figure 12.

Supplementary Figure 12 IGF1 mRNA expression following lentiviral infection with LV-pcIGF1.

Note: n = 3. Data are presented as mean ± SEM. *P < 0.05, **P < 0.01, ****P < 0.0001.

Supplementary Figure 13 IGF1 protein expression following lentiviral infection with LV-pcIGF1.

Note: Panel A: Western blot analysis of IGF1 in FLS. a: LV-ncIGF1; b: LV-pcIGF1. Panel B: Statistical results of IGF1 Western blot analysis in FLS. n = 3. Data are presented as mean ± SEM. *P < 0.05.

Supplementary Figure 14 Migration of FLS following lentiviral infection with LV-pcIGF1 and drug intervention.

Note: Panel A: Representative images of FLS migration following LV-pcIGF1 infection and drug intervention. a: LV-ncIGF1 + Placebo group, b: LV-ncIGF1 + LPS group, c: LV-ncIGF1 + LPS + TG group, d: LV-ncIGF1 + LPS + MTX group, e: LV-pcIGF1 + Placebo group, f: LV-pcIGF1 + LPS group, g: LV-pcIGF1 + LPS + TG group, h: LV-pcIGF1 + LPS + MTX group. Panel B: Statistical results of FLS migration in different groups following LV-pcIGF1 infection. n = 3. Data are presented as mean ± SEM. **P < 0.01.
